# Optimal Semiconductors for ^3^H and ^63^Ni Betavoltaics

**DOI:** 10.1038/s41598-019-47371-6

**Published:** 2019-07-26

**Authors:** Sergey I. Maximenko, Jim E. Moore, Chaffra A. Affouda, Phillip P. Jenkins

**Affiliations:** 1 Naval Research Laboratory, Washington, DC 20375 USA; 2 The George Washington University, Washington, DC 20052 USA

**Keywords:** Semiconductors, Nuclear energy, Batteries, Batteries, Electronic and spintronic devices

## Abstract

Betavoltaic power sources based on the conversion of radioisotope energy to electrical power are considered an appealing option for remote applications due to extended period of operation and high energy densities. However, to be competitive with other power sources, their efficiency must be increased. This can be done through optimization of the beta source and selection of the semiconductor absorber. This paper evaluates available on the market and developing wideband gap semiconductors as prospective absorbers with ^3^H and ^63^Ni sources. Simulation results indicate that among wide band gap materials 4H-SiC and diamond are two optimal semiconductors due to the combination of good coupling efficiencies with isotope sources and good electronic transport properties. Additionally, having good coupling efficiency, an ultra-wide bandgap, and the capability for both n- and p-type doping, c-BN is a promising material for betavoltaic applications.

## Introduction

**Figure 1:**
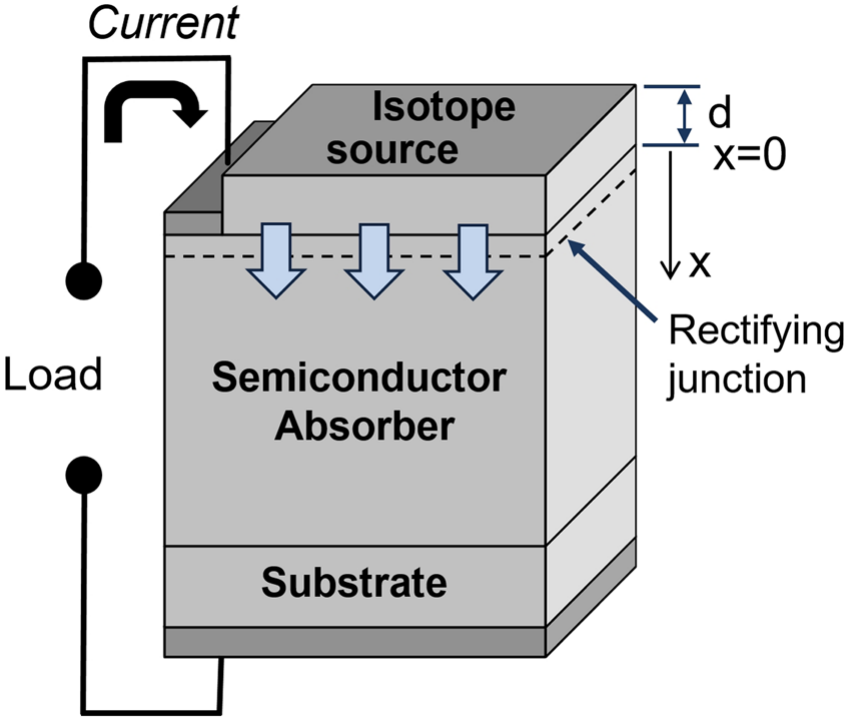
Schematic representation of betavoltaic device structure in planar configuration.

The direct conversion of particles emitted as a result of radioactive decay into electrical energy using semiconductors as absorber materials was proposed and demonstrated more than six decades ago^1^. Depending on the choice of the radioisotope, a nuclear energy converter is able to deliver electrical power output for several days or hundreds of years. The basic principle of direct nuclear-particle to electric energy conversion is to absorb nuclear particles in a semiconductor creating electron-hole pair charge carriers, and then separate those charge carriers in the semiconductor by use of a rectifying junction or carrier selective contact. This is shown schematically in Fig. 1. Generally, two types of decay particles, alpha or beta, can be used. Although alpha particles are more efficient to generate electron-hole pairs than beta particles, only beta particles are considered in this paper. Alpha particles generated from isotope decay due to their large mass create lattice damage in the semiconductor, rapidly degrading the electronic properties to such an extent that it will no longer function as an electronic device^2^.

The main advantages of betavoltaic sources over other conventional energy harvesting approaches are continuous operation and high energy density, but their utility is diminished by low output power (nano-, micro-Watt levels) and strict safety regulations. However, recent advancements in minimization of power consumption of electronic systems^3^ using small size electronic components and new energy efficient technologies have reintroduced an interest in radioisotope power sources.

Most common sources of beta-particles considered for betavoltaics^4–6^ are presented in Table 1. Selection is based on their availability, cost, half-life (1/λ), the average energy of the beta-particles (E_AVR_), and specific power (W_SPEC_). Considering that semiconductor lattice damage occurs when the beta-particle energy exceeds ~300 keV, the selection is narrowed to Tritium (^3^H), Nickel-63 (^63^Ni) and Promethium-147 (^147^Pm). While the ^147^Pm was an initial choice for the first betavoltaic batteries due to a high beta flux and average energy of beta particles, the relatively short half-life (2.6 years) make it less desirable as a long endurance power source. Conversely, ^3^H stands as a top choice due to high specific power, low shielding requirements, low cost and capability to store ^3^H in titanium forming Titanium Tritide (TiT_2_)_,_ where the typical ratio of ^3^H/Ti ~1.4–1.9^7^. Tritium can also be stored in organic compounds^8^. ^63^Ni is also an attractive option for specific applications due to its long half-life (100.2 years).

**Table 1:** The list of prospective isotope sources for a betavoltaic converters.

β-source	^3^H	^63^Ni	^147^Pm	^90^Sr/^90^Y
1/λ, [years]	12.3	100.2	2.6	28.8
E_AVR_, [keV]	5.7	17.4	62	195.8
E_MAX_, [keV]	18.6	66.9	225	546/2284
W_SPEC_, [W/kg]	324.9	5.79	412	160.3
Cost, $/Ci	~3.5–4	~4000	1000	—

The purpose of this study is to review the applicability of several commercially available and emerging wide bandgap semiconductors which can be or used for betavoltaics, along with conventional semiconductors such as Si and GaAs as benchmarks in combination with ^3^H and ^63^Ni isotope sources. This work provides a path on how to improve the power output of radioisotope energy converters by choosing of the optimal combination of semiconductor absorber and radioisotope source geometries. This is based on Monte Carlo simulations and an analytical model using the detailed balance limit originally derived for solar cells by Shockley and Queisser^9^.

## Simulation Approach

Simulation of the radioisotope energy converter power output consists of the modeling of the interaction between beta-particles (electrons) and parts of the converter. They include the semiconductor device structure (absorber) and the radioisotope source itself in basic planar geometry schematically presented in Fig. 1. In order to accurately calculate the energy dissipation in the semiconductor absorber it is important to use a full energy spectrum rather than the average energy of beta particles^10^. A Monte Carlo simulation routine is the most used approach, which calculates the correct output spectrum of beta particles from isotope source into the absorber by simulating the scattering and absorption in the source itself (self-absorption effect) and the energy distribution of particles in the absorber to give the resulting generation rate of electron-hole pairs. In this paper a two-dimensional (2D) Monte Carlo simulation program was used^11^ with  a computational routine adopted from ref.^12^. The modified Bethe energy dissipation function $\frac{dE}{ds}=7.85\times {10}^{4}\frac{\rho }{E}{\sum }_{i}\frac{{C}_{i}{Z}_{i}}{{A}_{i}}\,\mathrm{log}(1.116\frac{E}{{J}_{i}}+{K}_{i})$ is implemented to evaluate the particle energy loss^13^ where *C* is the atomic fraction, *Z is* the atomic number, *A* is the standard atomic weight, and *J* and *K* are the ionization energy and a correction term given in ref.^12^. Values of scattering cross sections of elements used in the simulation are derived from the ELSEPA database developed by NIST^14^. The Monte Carlo simulation assumes an isotropic emission of radiation from isotope sources^15^ and takes into account the energy spectra of the ^3^H and pure ^63^Ni beta sources (taken from ref.^16^), the material’s atomic properties, density, backscattering of particles and ionization potential. The absorber layer is divided into a mesh of cells and the distribution of energy absorbed by the semiconductor is output in the form of a 2D histogram. The generation rate can then be calculated by dividing the energy distribution by the ionization energy of the semiconductor material. Finally, the generation current is calculated from the generation rate using the equation ${J}_{GEN}=\frac{q}{W}\iint G(x,y)dxdy$ where *W* is the width of the semiconductor absorber in the simulation, and the integral is calculated by taking the summation of the generation rate per unit volume (*G*) in each cell of the simulation mesh multiplied by the cell’s area. Further Monte Carlo output data were used to analytically evaluate the power output of betavoltaic devices for two types of beta sources (^3^H and ^63^Ni) and selected semiconductor absorbers.

### Simulation results

A convenient way to evaluate the applicability of different semiconductor absorbers in combination with selected sources for betavoltaic applications is to estimate the efficiency and maximum delivered power output of the betavoltaic system. The efficiency of a betavoltaic system is composed of the efficiency of the source (*η*_*B*_), the coupling efficiency of semiconductor (*η*_*C*_) and the semiconductor conversion efficiency (*η*_*S*_) as was proposed by Olsen^17^.

**Figure 2:**
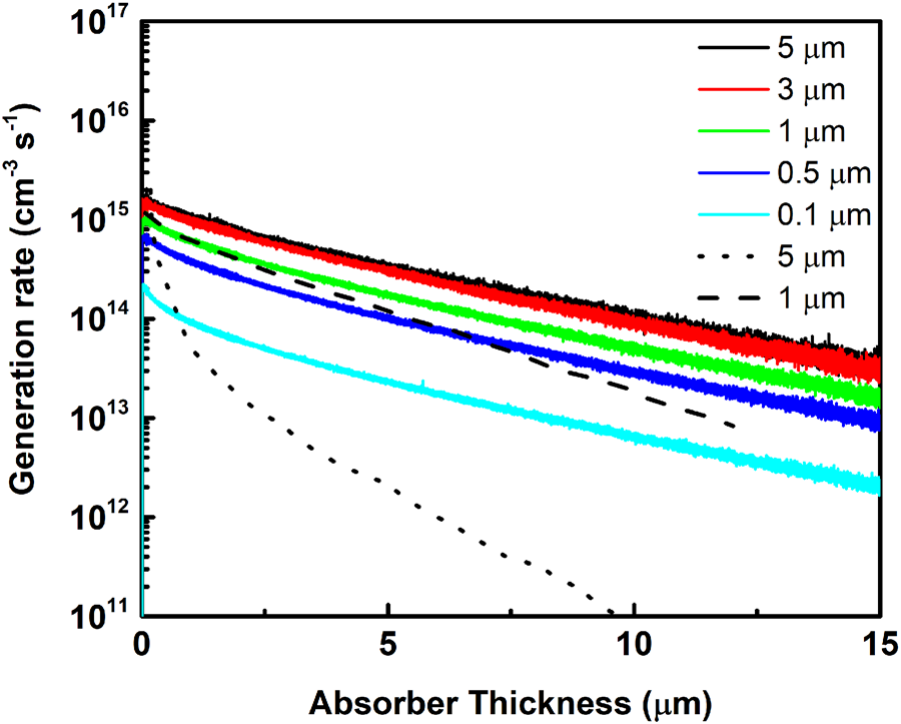
Distribution of excited carriers in 4H-SiC absorber coupled with ^63^Ni source (17.6% purity) and thickness of 0.1–5 µm. Dotted line from ref.^18^ and dashed line from ref.^10^ are shown for comparison.

In order to estimate the efficiency of the radioisotope source, defined as a ratio of the total emission power of beta particle decay within the source to the energy flux reaching the surface of the semiconductor from the source, it is important to find the optimal condition to deliver maximum power from a specific beta source. As a result of the self-absorption effect, a number of beta particles emitted by radioisotope are absorbed in the source material itself. Thinner layers are less impacted by the self-absorption mechanism, but also have less volume to store the isotope within, which results in reduced total emission. An example of a ^63^Ni (17.6% purity) source of various thicknesses coupled with 4H-SiC semiconductor absorber can be seen from Fig. 2. The figure presents the depth distribution of generated electron-hole particles versus absorber thickness. The increase of the source thickness (*d* in Fig. 1) leads to a saturation of electron-hole generation rate.

The results of the simulation are compared with published results for the same simulation conditions, which used Monte Carlo simulation routine (ref.^10^) and the direct chord method (ref.^18^). Similarly to ref.^10^, the simulation results presented in Fig. 2 do not match well to the direct chord method. Small differences between the results presented here and ref.^10^ can likely be explained by differences in boundary conditions and empirically determined constants. The model used here assumes that the simulated betavoltaic device area is large enough that edge losses are negligible. For a very small device (with an area on the order of a few square microns) the total generation rate will be smaller if these effects are accounted for. Additionally, empirical constants such as those found in the Bethe energy equation^13^ are not uniformly agreed upon and may differ between simulation programs.

**Figure 3:**
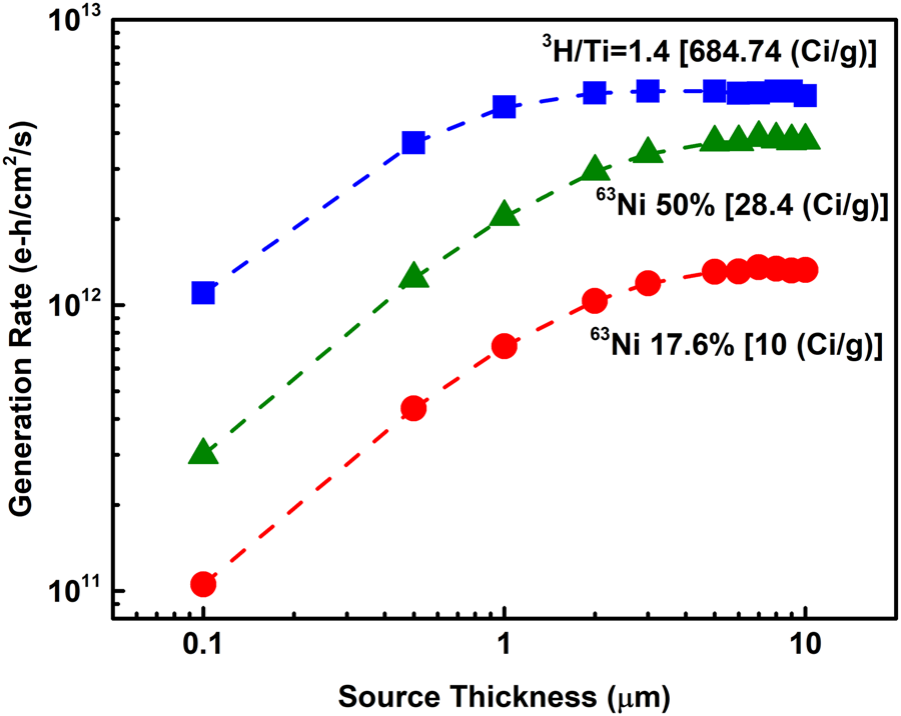
Total e-h generation rate of carriers calculated in 4H-SiC absorber versus thickness of ^3^H absorbed in Ti with ratio 1.4 and ^63^Ni with 17.6% and 50% purity films.

To find the optimal thickness of the beta-source a total generation rate was evaluated as a function of source thickness as presented in Fig. 3. Both ^63^Ni and ^3^H sources were evaluated. In the case of ^3^H a Titanium Tritide film was used, with the tritium absorbed ratio chosen to match ^3^H/Ti~1.4. At this concentration films are stable, and not brittle^19^. It can be seen that in the case of ^3^H at a thickness of above 1 µm the generation rate starts to saturate reaching a maximum total generation rate value at around 2 µm thickness. It corresponds to 0.764 Ci stored and up to a semiconductor incident power of 4.25 μW/cm^2^. For ^63^Ni (17.6% purity) saturation is observed at ~5 µm corresponding to 0.045 Ci and an incident power of 0.937 μW/cm^2^. The optimal thickness of the radioisotope source is not determined by the type of semiconductor absorber or the source purity but is strictly defined by the source physical properties such as radioisotope energy spectra and material density. This is demonstrated in Fig. 3 on an example of curves calculated for two ^63^Ni sources with different purities. Further, to evaluate the performance of semiconductors 5 µm thick ^63^Ni of 50% purity is used. It has 0.126 Ci stored and an incident power of 2.68 μW/cm^2^. Comparing a ^3^H/Ti (2 µm) source and a ^63^Ni source of 50% purity (5 µm), ^3^H/Ti has better efficiency (*η*_*B*_ = 0.16) in comparison with ^63^Ni (*η*_*B*_ = 0.13).

The betavoltaics semiconductor conversion efficiency (*η*_*S*_) is linked to the bandgap of the material (*E*_*GAP*_), similarly to the Shockley-Queisser limit for photovoltaics, and can be found through ${\eta }_{S}={V}_{OC}FF/\varepsilon$, where *V*_*OC*_ and *FF* are the open-circuit voltage and fill factor^17^ with $\varepsilon =2.8{E}_{GAP}+0.5$ [eV] ionization energy necessary to generate one e-h pair^20^. Open-circuit voltage can be defined as ${V}_{OC}=({k}_{B}T/q)\mathrm{ln}(({J}_{SC}/{J}_{0})+1)$ [V], where *k*_*B*_ is a Boltzmann constant, *T*-temperature, *q*-electron charge^21^. According to ref.^17^ the short-circuit current can be found as ${J}_{SC}=q(1-f)Q({P}_{SOURCE}/\varepsilon )$ [A/cm^2^] where *P*_*SOURCE*_ is the incident power of beta-source and particle backscatter coefficient (*f*). It should be noted that ref.^17^ uses average beta particle energy to calculate *P*_*SOURCE*_, while in this paper the full energy spectrum incident on the surface of the semiconductor is used and the short-circuit current is calculated by evaluating the generation current (*J*_*SC*_ = *J*_*GEN*_*Q*) as described previously in simulation approach section. A term, *Q*, represents collection efficiency of e-h pairs in the semiconductor. It is mostly defined by the minority diffusion length in a semiconductor material, approaching 1 if it is significantly longer than the maximum penetration depth of particles from the beta-source. The saturation current, *J*_0_, is estimated from ${J}_{0}=1.5\cdot {10}^{5}\exp (-{E}_{GAP}/{k}_{B}T)$ [A/cm^2^]^21^, which is an empirically simplified form of the full Shockley equation. The fill factor is found as $FF=[({V}_{OC}/{k}_{B}T)-\,\mathrm{ln}(({V}_{OC}/{k}_{B}T)+0.72)]/[({V}_{OC}/{k}_{B}T)+1]$^21^. From an analysis of the preceding expressions it can be seen that an increase in *E*_*GAP*_ leads to a decrease in *J*_0_ and increase in *V*_*OC*_, thereby increasing *η*_*S*_.

Prospective wide bandgap semiconductor materials along with conventional Si and GaAs as benchmarks used further for evaluation are listed according to the increase in the bandgap in Table 2. It contains their physical properties relevant to betavoltaics application and current technology status^22–32^. Si and GaAs were the first semiconductors used in betavoltaics^4^. Al_1-x_In_x_P lattice-matched to GaAs, specifically x = 0.47, and widely used for optoelectronic applications, offers the highest bandgap (*E*_*GAP*_~2.4 eV) among non-nitride III–V materials. Results on the use of this material in betavoltaics were published recently^33^. In_1-x_Ga_x_P is another III-V group direct wide band gap material. Its band gap energy varies with a composition (*E*_*GAP*_~1.6–2.2 eV). It is typically grown on a GaAs substrate using a lattice matched concentration of x = 0.5, corresponding to *E*_*GAP*_ = 1.9 eV. This material is widely used in solar cell technology and was reported in use for tritium based betavoltaics^34^. Wide bandgap 4H-SiC is an attractive material due to relative maturity of the technology and low dislocation density^22^. 4H-SiC betavoltaic devices^35,36^ are commercially available from Widetronix Inc^37^. GaN and GaN-based alloys also get a lot of attention as potential betavoltaics absorbers due to their wide bandgap and availability of relatively large size substrates^38–40^. The best crystal quality single crystalline GaN up to 2” boules have been prepared by the hydrogenated vapor phase epitaxial growth (HVPE), however larger size hetero-epitaxial grown GaN on large silicon wafers is also available. The larger bandgap Al-rich Al_1-x_Ga_x_N ternary semiconductor system is an attractive option, though the problem of crystal quality in Al-rich alloys due to degradation of minority diffusion length and poor n-type doping are serious problems. Another novel ultra-wide bandgap material is β-Ga_2_O_3_, which is also considered for betavoltaics. One serious drawback of β-Ga_2_O_3_ is the lack of suitable shallow acceptor dopants. Several groups reported the use of diamond as absorber material for betavoltaics (for example ref.^41,42^). The primary limitations of diamond are the small size of substrates and limited n-type conductivity. However, few reports on working bipolar devices have been demonstrated^43,44^. As can be seen from Table 2, semiconductors with bandgaps larger than 2H-GaN have poor abilities or cannot be doped to both types of electrical conductivity. The exception is the zinc blend structure, cubic boron nitride (c-BN). This semiconductor is currently in the very early stages of development. However, several properties such as an ability to be doped n- and p-type, the widest bandgap among listed semiconductors make this material very attractive for potential future electronic applications including betavoltaics^32^.

**Table 2:** The list of selected semiconductors with present state-of-the-art status.

Material	Si	GaAs	In_0.49_Ga_0.51_P	Al_0.53_In_0.47_P	4H-SiC	2H-GaN	Al_0.5_Ga_0.5_N	β-Ga_2_O_3_	Al_0.75_Ga_0.25_N	Diamond	2H-AlN	c-BN
Bandgap, [eV] Direct (D) Indirect (I)	1.12, I	1.42, D	1.9, D	2.4, I	3.23, I	3.44, D	4.52, D	4.9, D	5.25, D	5.5, I	6.05, D	6.4, I
Density, [g/cm^3^]	2.33	5.32	4.4	2.4	3.21	5.88	4.68	6.1	3.9	3.52	3.26	3.48
Substrate quality (dislocations per cm^2^)	0	10^3^	10^3^	10^3^	10^2^	10^4^	10^4^	10^4^	10^4^	10^5^	10^4^	—
Minority carrier diffusion length (µm)*	>40	~6	—	—	~12	~1	<1	0.4	<1	~40	—	—
Substrate diameter (inch)	>8	6	6 (on GaAs)	6 (on GaAs)	8	8 (on Si)	2	4	2	1	2	<0.2
Limitations	—	—	—	—	—	—	Poor p-type	No p-type	Poor p-type	Poor n-type	No p-type	Substrate size

*Reported minority carrier diffusion length for p-doped material. Values for Al_0.5_Ga_0.5_N, β-Ga_2_O_3_, Al_0.75_Ga_0.25_N, 2H-AlN are for n-doped material.

**Figure 4:**
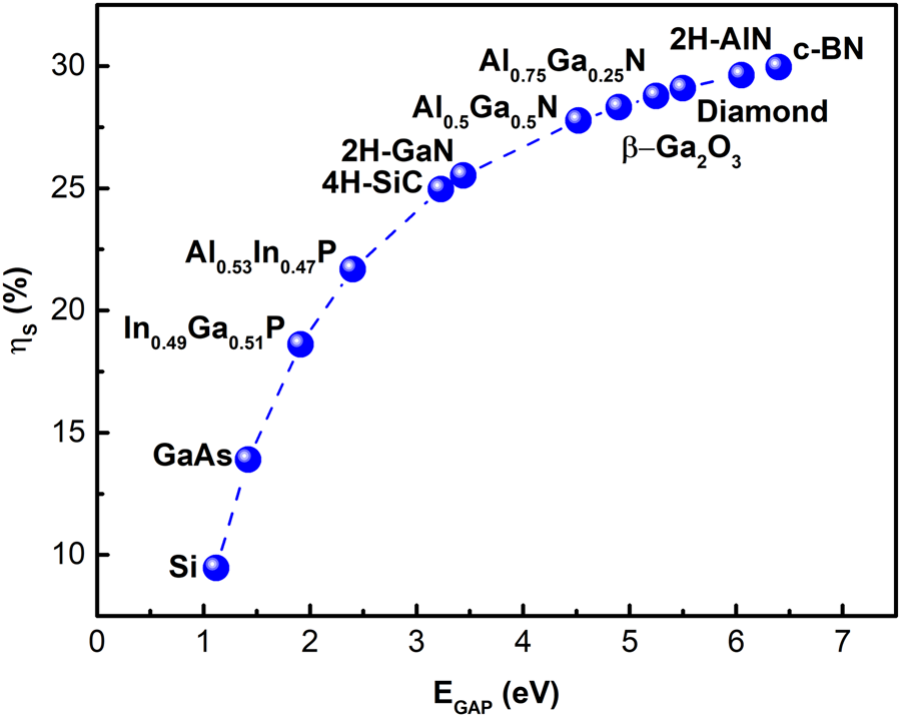
The semiconductor conversion efficiency (*η*_*S*_) in Shockley-Queisser approximation versus semiconductor bandgap calculated using ^63^Ni 5 µm (50% purity) source.

A necessary condition for betavoltaic energy conversion, is the formation of a rectifying junction (diode) to collect generated electron-hole carriers. There are two ways to form a rectifying junction, Schottky barriers or p-n doped junctions, however the latter is superior option due to better collection efficiency (Q) of generated charge carriers. In order to directly compare these semiconductors for betavoltaics application it is convenient to apply the Shockley-Queisser approximation, which assumes only radiative recombination (*Q* = 1) with the conventional device geometry shown in Fig. 1. The calculated semiconductor converting efficiencies (*η*_*S*_) are presented in Fig. 4. It gradually increases with a material gap and reaches ~30% for widest bandgap materials.

**Figure 5:**
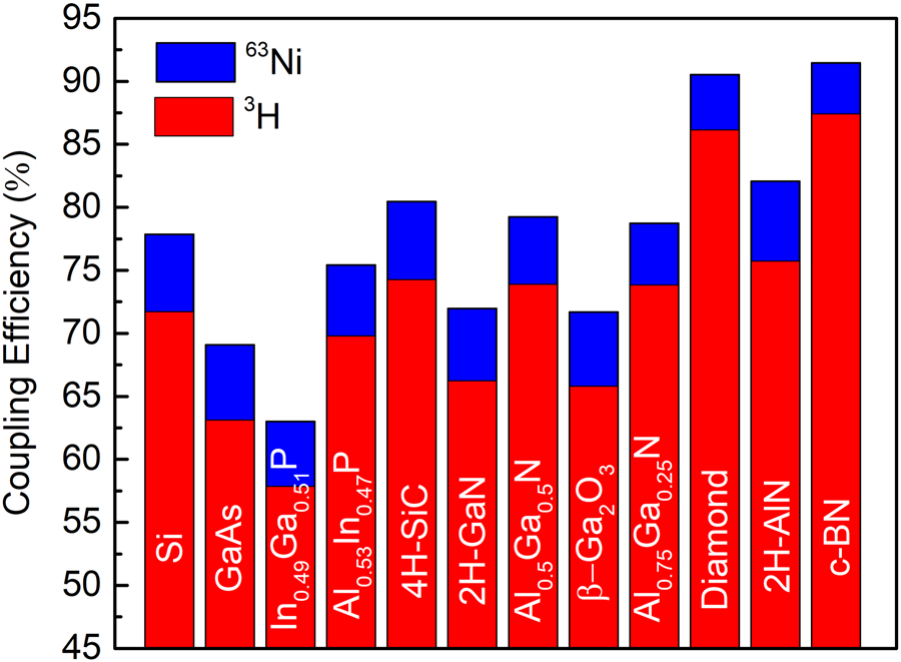
The semiconductor coupling efficiency (*η*_*C*_) in Shockley-Queisser approximation calculated for optimized ^63^Ni with 50% purity and ^3^H/Ti~1.4 sources.

As previously mentioned, another important parameter contributing to betavoltaic conversion efficiency is the coupling efficiency (*η*_*C*_) of the semiconductor with the source ${\eta }_{C}=(1-f)Q$. This depends on particle backscatter coefficient (*f*) and collection efficiency of e-h pairs in semiconductor (*Q*). Since, the backscatter coefficient in general depends on the energy of incident electrons^45^, rather than estimating backscatter coefficient for average energy of beta particles, a more accurate approach is to use a ratio of total incident energy flux to energy flux of backscattered electrons. Both parameters can be extracted from Monte Carlo simulation results. The calculated coupling efficiencies (*η*_*C*_) of semiconductors for both types of sources are presented in Fig. 5. Analysis of calculated data shows that ^63^Ni has better coupling efficiency than a tritium source, in the range of ~10%, with all semiconductors’ absorbers. This is due to the higher average energy of ^63^Ni (17.4 keV) in comparison to ^3^H (5.7 keV). According to the mean free path expression^13^ the rate distance between scattering events increases with increasing electron energy due to the energy dependence of the electron scattering cross section. Also, another observation is that Ga containing compounds such as GaAs, GaN, Ga_2_O_3_ and In_1-x_Ga_x_P have significantly lower coupling efficiency in comparison with other semiconductors presented. This is due to the large effective atomic numbers of those materials. On the other hand, diamond and c-BN have the best coupling with both isotope sources, due to low effective atomic numbers of those semiconductors. In general, it is a straight correlation between effective atomic number and backscattering coefficient^46^, and an increase in the energy loss through backscattering with increasing effective atomic number of semiconductor.

**Figure 6:**
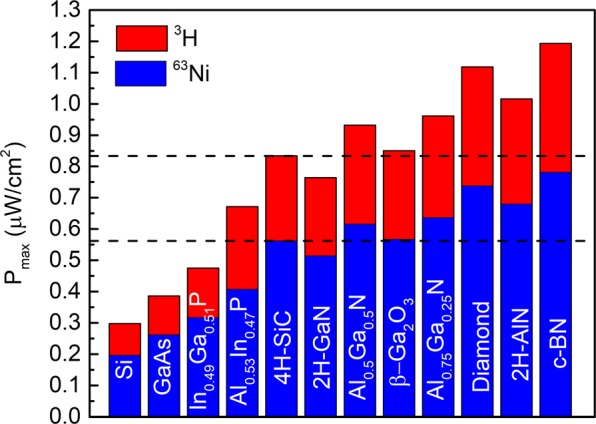
Maximum output power of semiconductor absorbers in Shockley-Queisser approximation for optimized ^63^Ni with 50% purity and ^3^H/Ti~1.4 sources. Dashed lines show *P*_*max*_ of 4H-SiC.

The maximum power output of the betavoltaic device similar to a photovoltaic device can be defined as ${P}_{\max }={V}_{OC}{J}_{SC}FF$^21^. The results of calculations for semiconductors coupled with both sources are presented in Fig. 6. The impact of semiconductor and isotope coupling efficiency on power output is quite pronounced. Thus, GaN with larger bandgap (*E*_*GAP*_ = 3.44 eV) than 4H-SiC (*E*_*GAP*_ = 3.23 eV) has less maximum power output, due to an impact of inferior coupling efficiency. Another Ga containing semiconductor, β-Ga_2_O_3_ with significantly larger bandgap (*E*_*GAP*_ = 4.9 eV) than 4H-SiC has a comparable power output as a result of a large backscatter fraction of beta-particles. Also, it can be clearly seen that 2H-AlN as well is not the best option for betavoltaic applications. Diamond (*E*_*GAP*_ = 5.5 eV) and c-BN, (*E*_*GAP*_ = 6.4 eV), having the largest power outputs among all reviewed here semiconductor absorbers, are the optimal choices. This is a result of their large bandgaps and efficient coupling with both radioisotope sources.

**Figure 7:**
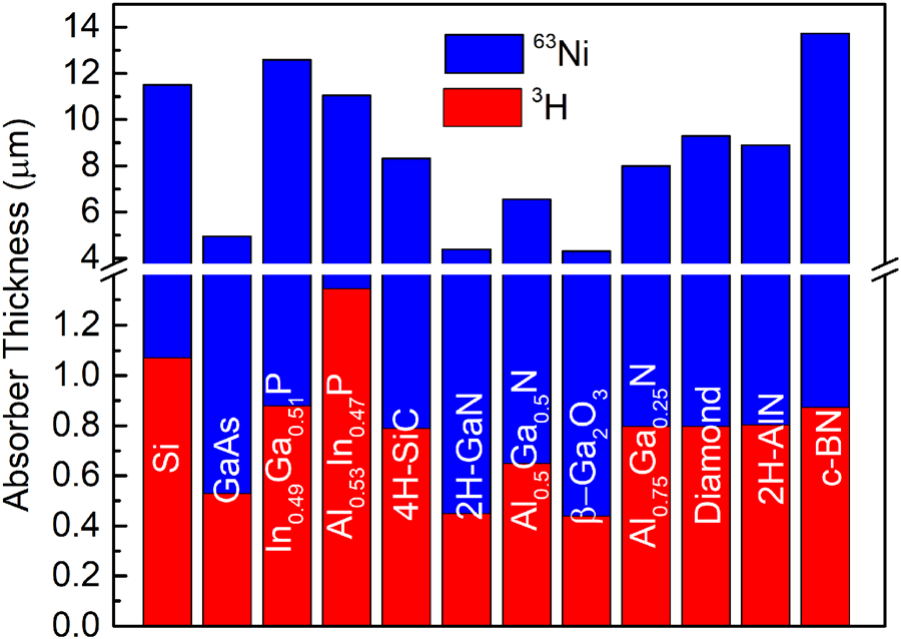
Calculated absorber thickness required to deliver 87% of maximum output power in Shockley-Queisser approximation for optimized ^63^Ni with 50% purity and ^3^H/Ti~1.4 sources.

Finally, the absorber thickness required to effectively absorb the majority of beta-particles is of interest for the betavoltaic design. It can be estimated by introducing an effective absorber thickness at which point 87% of maximum output power is collected to analogy with penetration depth that describes the decay of electromagnetic waves inside of a material. The results of these calculations are presented in Fig. 7. The majority of absorbers coupled with ^3^H requires less than 1 µm of thickness to effectively absorb beta-particles, while for a ^63^Ni source the required thickness is in excess of 5 µm. The necessary absorber thickness to absorb beta-particles depends on the density of semiconductors and the energy of the beta particles from the source. Materials with a high density such as Ga containing compounds require thinner layers than low-density materials.

A required absorber thickness puts restrictions on the type of semiconductor which can be used with a specific isotope. Thus in a simple approximation, similar to solar cells, a collection efficiency of e-h pairs in the semiconductor (*Q*) depends on the minority carrier diffusion length (*L*) as $Q=1-\,\tanh (x/L)$, where *x* is the distance from depletion region. Longer diffusion lengths generally result in better performance. The analysis of structures based on the voltaic effect, for example solar cells, shows that the peak efficiency for a BSF (back surface field) cell structure is at a condition where the minority carrier diffusion length is two times longer than the base thickness (for example ref.^47^). This ratio can be applied to a betavoltaic system to obtain maximum collection efficiency. Following this rule, it can be seen that Si, 4H-SiC and diamond are the materials of choice to use with an ^63^Ni source. Indirect bandgaps of those materials provide longer diffusion lengths to fit those requirements compared to other listed in Table 2 direct bandgap semiconductors. In addition to those materials GaAs, 2H-GaN, and to a lesser extent GaN-based alloys can be candidates for ^3^H based betavoltaics.

It should be noted that an additional benefit of using a ^3^H source is the cost factor. Due to the lower energy of particles emitted from ^3^H, thinner absorbers are required. Thinner absorbers are cheaper, so the most cost-effective combination of source and semiconductor materials can be achieved with ^3^H. Furthermore, materials with lower diffusion lengths may be useable with ^3^H due to the thinner absorber requirement.

Among the evaluated semiconductors for ^3^H and ^63^Ni betavoltaics, two wide bandgap semiconductors, 4H-SiC and diamond stand up as the optimal choice materials due to good coupling efficiencies with isotope sources and long minority carrier diffusion lengths matching absorber thicknesses requirements to effectively absorb the majority of beta-particles. One more interesting candidate for forthcoming betavoltaic applications is the indirect bandgap semiconductor c-BN. However, this material is still in an early development stage.

## Conclusion

In this work, available on the market and developing wide bandgap semiconductors were evaluated for use as absorbers in planar betavoltaics structures with ^3^H and ^63^Ni radioisotope sources. Numerical Monte Carlo simulations incorporating the full energy spectra of radioisotopes, including self-absorption effects, and an analytical model in Shockley-Queisser approximation were utilized to analyze the betavoltaic performance of materials. The results suggest 4H-SiC and diamond as optimal materials for ^3^H and ^63^Ni betavoltaics. 4H-SiC has long diffusion lengths, is available in large substrates, and can be doped both n- and p-type. At the same time diamond potentially could deliver ~34% increase in output power density (^3^H source) (Fig. 6) in comparison with 4H-SiC and could be considered as the next generation betavoltaic material. However, n-type doping issues need to be resolved^48^. Another promising candidate for future betavoltaics is the indirect semiconductor c-BN, due to its ability to be doped both n- and p-type, with great coupling efficiency, and is one of the widest bandgap materials among semiconductors today. Gallium-containing direct bandgap compounds such as GaAs, GaN, Ga_2_O_3_ and In_1-x_Ga_x_P have low coupling efficiency with radioisotope sources and short diffusion length of minority carriers (except GaAs) and thus expected to be less efficient choice for ^3^H and ^63^Ni betavoltaics.
